# Harnessing AI for Antimicrobial Peptide Innovation against Multidrug Resistance

**DOI:** 10.1021/jacsau.5c01520

**Published:** 2026-02-02

**Authors:** João P. F. Pimentel, Raquel M. Quigua Orozco, Samilla Beatriz de Rezende, Lucas Lima, Marlon H. Cardoso

**Affiliations:** 1 S-Inova Biotech, Programa de Pós-Graduação em Biotecnologia, Universidade Católica Dom Bosco, Campo Grande-MS 79117900, Brazil; 2 Programa de Pós-Graduação em Ciências Ambientais e Sustentabilidade Agropecuária, Universidade Católica Dom Bosco, Campo Grande-MS 79117900, Brazil

**Keywords:** artificial intelligence, antimicrobial peptides, machine learning, deep learning, peptide-based drugs

## Abstract

Antimicrobial resistance (AMR) poses a critical global health threat, demanding innovative strategies for drug discovery. Antimicrobial peptides (AMPs) represent promising alternatives, yet traditional experimental identification is limited by cost and scalability. Advances in artificial intelligence (AI), particularly machine learning (ML) and deep learning (DL), have transformed AMP discovery by enabling the accurate prediction, design, and optimization of novel candidates. This perspective highlights recent progress in AI-driven approaches, including predictive models and generative models, which accelerate large-scale peptide screening and functional annotation. We further emphasize the integration of multiomics data and the potential role of emerging technologies, such as quantum computing (QC), in overcoming computational bottlenecks for peptide design. Together, these approaches promise to expand the therapeutic landscape, paving the way toward next-generation peptide-based antimicrobials capable of circumventing resistance mechanisms and addressing urgent clinical needs.

## Graphical abstract

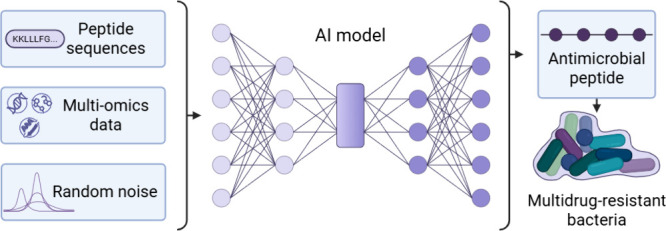

## 1 Introduction

As recognized and widely reported by several governmental, nongovernmental, and intergovernmental organizations, including the World Health Organization (WHO), the Food and Agriculture Organization of the United Nations (FAO), and the Centers for Disease Control and Prevention (CDC), antimicrobial resistance (AMR) is on track to become the globally leading cause of death in the coming decades.^1,2^ Currently, approximately 1.2 million annual deaths are attributed to antimicrobial resistance, and it is estimated that these microorganisms could cause up to 10 million deaths by 2050.^3^ The slow pace of new antibiotic discovery, coupled with the rapid emergence of AMR, has significantly intensified the challenge of developing effective therapies.^4,5^ The shortage of new antibiotic candidates underscores the requirement for effective experimental methods, allowing for the rapid identification of the microorganisms and their antibiotic susceptibility profiles, as well as the elucidation of the molecular mechanisms of resistance.^6^ From this perspective, new alternatives are needed to prospect for new classes of antimicrobials.^7^

Bioactive peptides have become attractive due to their diverse structures and broad spectrum of bioactivities, particularly their antimicrobial properties.^8,9^ Antimicrobial peptides (AMPs) are ubiquitous in various organisms. They can belong to different molecular classes and play a significant role in the innate immune system, exhibiting diverse activities and targeting a wide range of pathogens, including viruses, fungi, bacteria, protozoa, and even neutralizing toxins.^10^ The pharmacodynamic differences between conventional antibiotics and AMPs underscore their potential as a promising alternative for combating AMR. Unlike traditional antibiotics, AMPs exhibit unique mechanisms of action that reduce the likelihood of resistance development and mutagenic induction, making them a valuable tool in the fight against resistant pathogens.^7^ Traditional strategies in AMP design leverage a wide array of computational tools, as demonstrated across multiple studies.^10–12^ *De novo* modeling approaches generate novel AMP sequences by systematically using amino acid preferences to achieve critical properties, such as structural stability and amphipathicity. Linguistic frameworks employ formalized “grammars” based on amino acid motifs, enabling targeted and rule-based sequence design that captures functional patterns. Pattern insertion methods utilize curated amino acid motifs mined from extensive public databases to enhance peptide activity and specificity.^10,11^ Meanwhile, evolutionary algorithms, including genetic and multiobjective optimization techniques, iteratively apply mutation, crossover, and selection to refine peptide fitness for antimicrobial efficacy.^10^

The functionality of a peptide is significantly influenced by its amino acid sequence and the structural scaffold. These variables are critical factors that researchers must consider when designing peptides with specific activities.^13^ To guide the development process, tools such as hydrophobicity scales and secondary structure propensity tables are used, as they provide essential insights into how amino acid properties and peptide structures interact to achieve the desired functionality. By carefully evaluating these parameters, researchers can optimize peptide design to enhance efficacy and specificity for their intended applications.^13^

However, traditional methods face limitations due to the vast number of possible peptide sequences. For a peptide composed of only the 20 naturally occurring amino acids, the sequence possibilities equal 20̂*n*, where *n* is the peptide sequence length. This means that even a short peptide of 10 residues has over 10 trillion possible amino acid conformation possibilities, leaving a vast number of sequences unexplored.^13^ An alternative approach involves machine learning (ML) models, an artificial intelligence (AI) framework that supports effective decision-making by learning patterns from large training datasets. Numerous ML algorithms have been applied to develop innovative peptide sequences.^10,13^

By leveraging large datasets, ML algorithms can identify patterns and predict the activity, toxicity, and stability of AMPs, significantly reducing the time and cost associated with traditional experimental methods.^14^ Deep learning (DL), a specialized area within ML, employs networks with multiple processing layers that learn to represent data at various levels of abstraction. These multilayered neural networks are highly effective in analyzing large datasets and making complex decisions. By using the back-propagation algorithm, DL enables these models to adjust their internal parameters, enhancing the representation of information layer by layer. This capability has led to significant advances in fields such as speech recognition, visual object recognition, object detection, and applications in drug discovery.^15–17^

In this perspective, we will explore the most important neural network models and AI architectures designed to tackle the growing problem of AMR. By diving into recent breakthroughs, we aim to show how these technologies can help speed up the discovery and optimization of new AMPs and enhance treatment approaches, offering hope in the fight against resistant infections.

## 2 Development of AI Models for AMP Discovery

AI refers to the creation of “intelligent” systems capable of performing tasks typically requiring human intelligence, including learning, problem-solving, and decision-making.^15,16^ These models offer significant advantages by accelerating the AMP discovery process, allowing for the rapid screening and optimization of peptide candidates with desired antimicrobial properties.^14^ ML is a branch of AI that focuses on creating algorithms that learn from raw data to perform specific tasks. These tasks typically include classification, regression, clustering, and pattern recognition within large datasets.^15,16^

To address the challenge of AMR, researchers have successfully applied AI models to search for new AMPs (Figure[Fig fig1]). Advances in graphics processing unit (GPU) technology have significantly increased the speed and precision of ML model training, accelerating the discovery of peptides with desired activities.^18^ We can cite three main strategies for using AI to develop new AMPs, including (I) training models to generate new sequences, (II) mining potential peptides from large datasets,^19^ and (III) optimizing sequences to aim for enhanced efficacy and reduced toxicity.^20^

**Figure 1:**
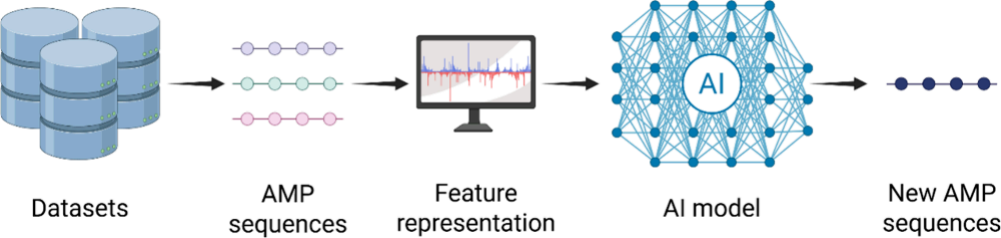
Flowchart representing the process of identifying and generating new AMP sequences using AI. The input data consist of known AMP sequences extracted from databases, which undergo feature extraction steps. This information is processed by an AI model, resulting in the prediction of new AMP sequences. This figure was created with BioRender.com.

Before making predictions and enabling the discovery of new AMPs, ML models must first be trained by adjusting their parameters to optimize performance.^21^ For efficient training and the development of highly accurate models, AI relies on extensive datasets of high-quality, accurate information, coupled with substantial computational resources. However, the collection of such data has been a limiting factor.^22^ The accumulation of data on small molecules and proteins has led to the development of extensive databases containing molecular sequences, structures, and properties.^23^ The challenge of gathering high-quality data has become a limiting factor in the application of AI for peptide drug development.^19^ Although there are publicly available datasets for protein informatics with labeled protein activity, their size is often limited. Notwithstanding this, the dataset size can become smaller after filtering based on how the activity and properties were measured. This information tends to be dispersed and fragmented,^24^ and it is becoming even more scarce for properties such as nonantimicrobial activity-labeled data.^18^

The absence of standardized activity thresholds and consistent definitions of minimum inhibitory concentration (MIC) across different studies complicates the comparison and reproducibility of AMP prediction models. This issue has been highlighted in multiple reviews and benchmarking studies, which emphasize the urgent need for adopting unified standards and benchmark datasets to enable rigorous evaluation and comparison of models.^20,21^ In response, some recent models have integrated explicit MIC prediction as part of their output, aiming to provide more quantitative and comparable assessments of peptide activity.^20^ Nonetheless, variability in experimental protocols and reporting practices remains a significant barrier, limiting the direct comparability of results and underscoring the necessity for standardized experimental and data curation procedures.^11,20,21^

Additionally, the heterogeneity of the data formats and sources further complicates model development. Integrating such diverse information requires appropriate strategies for molecular representation and standardized input formats.^23^ In this context, peptides can be encoded and characterized through various methods that capture their structural and functional properties.^18^ A common approach involves representing peptides by their primary amino acid sequences, which can be encoded as strings or matrices, where each amino acid is assigned a unique vector, such as through one-hot encoding. While straightforward, one-hot encoding lacks information about amino acid properties and similarities.^18^

Most feature representations have focused on peptides composed of the 20 natural amino acids, often excluding noncanonical or chemically modified amino acids during training. Consequently, these models may struggle to generalize to nonstandard peptides.^20^ Advanced representations, including topological fingerprints, preserve structural details of each residue, offering a more comprehensive depiction.^18,22^ Despite the rise of modern techniques that extract functional and structural insights from sequence data, traditional methods like one-hot encoding and physicochemical descriptors remain relevant and applicable.^22^

Once the features are computationally represented, the next step is to leverage them for predictive modeling. At this stage, ML models undergo training in which parameters are optimized to maximize the predictive performance. Most current AI approaches for AMP prediction rely on supervised learning, which uses labeled data validated through experimental evidence, including toxicity assays, structural annotations, or wet-lab-confirmed AMP sequences.^21,25^ Supervised learning is designed to map inputs to outputs with high accuracy, often surpassing human-level performance once training is complete.^25^ This capacity to consistently associate new input examples with the correct outputs explains why supervised learning is particularly effective for predictive tasks.

### 2.1 Predictor Models

#### 2.1.1 AMP Predictors

LMPred applied convolutional neural networks (CNNs) after generating contextualized embeddings of amino acid sequences through pretrained language models. This two-step approach enabled the CNN classifier to distinguish AMPs from non-AMPs with a predictive accuracy of 93.33% (Table 1).^26^ CNNs are particularly effective in handling grid-structured representations, such as protein inter-residue distance maps, because they exploit parameter sharing and sparse local interactions, reducing computational cost while allowing the processing of inputs with variable sizes. This distinctive architectural design enables equivariant representations and overcomes the key limitations of traditional feedforward networks. Consequently, CNN-based approaches have been widely adopted in practical applications, often relying on large and complex architectures that incorporate multiple variants and millions of parameters to capture relevant spatial patterns in peptide-related data.^27,28^ Within this framework, LMPred predicts antimicrobial activity directly from contextualized sequence embeddings generated by pretrained protein language models, without relying on manually engineered physicochemical descriptors. A limitation of LMPred is its dependence on the representational power of the embeddings since CNNs primarily capture local motifs and may fail to integrate long-range dependencies if those are not encoded in the embeddings themselves. This indicates that its performance is strongly tied to the quality of upstream language models; therefore, may influence the performance of the identified peptides in the context of AMR.^26^

**Table 1:** Summary of Models by Purpose, Performance, and Methodology^a^

**model/study**	purpose	key findings/performance	architecture/methodology	ref.
LMPred	AMP prediction	achieved 93.33 and 88.26% accuracy on two datasets, outperforming prior models	uses pretrained protein language models to create embeddings fed into CNN classifier	^26^
AMPlify	AMP prediction	achieved >90% accuracy, identified novel AMPs active against multidrug-resistant bacteria	Bi-LSTM combined with ATT, including multihead-scaled dot-product attention and context attention layers	^29,30^
AI4AMP	AMP prediction	achieved approximately 90% precision, outperforming current AMP predictors on external testing	CNN model trained on balanced AMP/non-AMP dataset	^60^
TP-LMMSG	predict antiviral, anticancer, and AMPs	outperforms current state-of-the-art models while remaining robust with imbalanced data and achieving 7x faster preprocessing.	GNN combined with optimized LM and peptide graph representations derived from structural predictions	^34^
sAMPpred-GAT	AMP prediction	graph-based modeling of residue-level interactions using peptide structural information predicted by trRosetta	GAT operates on peptide graphs constructed from predicted residue contact information and sequence-derived features	^33^
PGAT-ABPp	ABP prediction	integration of sequence embeddings and structural features predicted by AlphaFold2	hybrid framework combining PLM embeddings with GATs	^32^
EIPpred	MIC predictor	provides MIC prediction for inhibitory peptides against E. coli with low computational complexity	RF regression trained on experimentally determined MIC values	^36^
BERT-AmPEP60	MIC predictor	provides reliable and explainable predictions of peptide antimicrobial activity, enabling informed candidate selection	fine-tuned ProtBERT via transfer learning on AMP datasets and trained to predict MIC values using a regression model	^39^
Almotairi et al.^37^	hemolysis predictor	accurate classification of hemolytic and nonhemolytic peptides using sequence information, enabling early host-toxicity filtering	hybrid deep learning model combining transformer layers for global sequence context with CNNs for local information extraction	^37^
ToxGIN	toxicity predictor	enhances peptide toxicity assessment by capturing spatial residue interactions not accessible to sequence-only predictors	GIN operates on graphs derived from predicted peptide structures	^38^
VAMPr	AMR prediction	high accuracy (mean 91.1%), confirms known and novel resistance variants; validated on external datasets	gradient boosting trees (XGBoost) with gene ortholog-based variant features and nested cross-validation	^43^
Das et al.^50^	generate and screen novel antimicrobial peptides with broad potency and low toxicity	discovered two potent, low-toxicity AMPs effective against multidrug-resistant pathogens within 48 days	VAE with DL classifiers and molecular dynamics simulations	^50^
QMO	optimize molecules for better drug-likeness, solubility, binding affinity, and toxicity reduction	outperforms baselines by 15%, improves SARS-CoV-2 inhibitor affinity, and reduces AMP toxicity with 72% success	VAE with BO, Gaussian sampling, predictors, and evolutionary algorithms for molecule optimization	^51^
MOQA	design nonhemolytic antimicrobial peptides using quantum annealing	discovered nonhemolytic AMPs with a 1/100,000 hit rate, outperforming BO and RNNs	bVAE, quantum annealer, factorization machine, multiobjective optimization loop	^52^
Multi-CGAN	design AMPs with multiple optimized properties	generated peptides with strong activity, broad-spectrum potential, and improved physicochemical traits	Conditional GAN with multiproperty conditioning, deep generative framework for sequence design and evaluation	^54^
Ma et al.^8^	identify AMPs from human gut microbiome using AI	identified 2349 candidate AMPs; 181 synthesized showed >83% antimicrobial activity, low homology to known AMPs	combination of LSTM, ATT, and BERT neural networks	^8^
Santos-Júnior et al.^59^	identify AMPs across global microbiomes using ML	discovered 863,498 AMP candidates, multiple peptides showed potent antimicrobial activity *in vitro* and *in vivo*	integrated metagenomic data with deep learning approaches	^59^
panCleave	predict protease cleavage sites	identifies encrypted AMPs in modern and archaic human proteins with *in vitro*/*in vivo* activity	RF trained on pan-protease cleavage site data for computational proteolysis of human and archaic proteomes	^63^
APEX	predict antimicrobial activity and discover peptides effective against Gram-positive/-negative bacteria	APEX accurately predicts species-specific AMPs, discovers selective and broad-spectrum EPs with high validation success	RNN with GRU, layer normalization, and two-layer attention for peptide sequence feature extraction	^64^
Guan et al.^65^	explore global animal venoms to discover novel therapeutic peptides	identified anticancer, neuroactive and AMPs candidates, highlighting venom diversity as a drug discovery source	integrated APEX DL predictor with multiomics and computational screening to annotate venom peptides and assess therapeutic potential	^65^
APEX_GO_	optimize AMPs from extinct organisms for enhanced antibiotic activity	achieved 85% experimental hit rate, improved antimicrobial efficacy, peptides outperform some antibiotics *in vitro* and *in vivo*	transformer-based VAE with Gaussian process-based BO over latent space and trust regions	^66^

a Abbreviations: AMP (antimicrobial peptide), CNN (convolutional neural network), LSTM (long short-term memory), Bi-LSTM (bidirectional long Short-term memory), ATT (attention), GNN (graph neural network), LM (language model), GAT (graph attention network), ABP (antibacterial peptide), PLM (protein language model), MIC (minimum inhibitory concentration), BERT (bidirectional encoder representations from transformers), RF (random forest), GIN (graph isomorphism network), AMR (antimicrobial resistance), VAE (variational autoencoder), DL (deep learning), QMO (query-based molecule optimization), SARS-CoV2 (severe acute respiratory syndrome coronavirus 2), BO (Bayesian optimization), bVAE (bidirectional variational autoencoder), MOQA (multiobjective optimization by quantum annealing), RNN (recurrent neural network), GAN (generative adversarial network), CGAN (conditional generative adversarial network), EP (encrypted peptides), GRU (gated recurrent unit).

As an alternative to its limitation, recurrent neural networks (RNNs) are ideal for processing sequential data such as natural language and protein sequences. RNNs maintain an internal state that is updated as input sequences are processed, enabling them to capture interactions between distant elements.^27,28^ Variants of RNNs, like long short-term memory (LSTM) and gated recurrent units (GRU), use gating mechanisms to manage short- and long-term dependencies in sequences, playing a key role in fields such as natural language processing (NLP) and bioinformatics. In this context, RNN-based models are well suited for protein sequence representation learning, as they capture sequential dependencies and long-range interactions across peptide chains while modeling correlations between amino acid residues across multiple dimensions.^27,28^ As an example of the use of RNNs in the realm of AMPs, AMPlify employed a bidirectional long short-term memory (Bi-LSTM) network with an attention (ATT) mechanism and an ensemble strategy to predict AMPs (Table 1). By reading sequences in both forward and reverse directions, the Bi-LSTM captures long-distance dependencies across amino acids, while the attention layer emphasizes informative regions and the ensemble reduces prediction variance.^29^ These architectural elements allowed the model to achieve accuracy above 90%. However, Bi-LSTM models are sensitive to dataset composition, particularly class imbalance and sequence redundancy, which may lead to inflated performance estimates. They also require longer training times compared with transformer-based architectures, which have become state of the art in other domains of sequence analysis.^29,30^

Another innovative approach is geometric deep learning (GDL) that focuses on processing structured data in non-Euclidean spaces, making it ideal for handling complex data such as the three-dimensional structures of proteins and peptides.^28,31^ Graph neural networks (GNN), a type of GDL, operate on graph-based, non-Euclidean data structures and are used for problems such as clustering, link prediction, and node classification. GNNs have emerged as powerful tools in drug discovery and protein bioinformatics because they naturally accommodate the relational and spatial characteristics of biomolecules. By representing proteins as graphs derived from sequences or residue–residue contacts, these models can learn features directly relevant to a given task, reducing the dependence on manually designed descriptors. In addition, advances in protein structure prediction have enabled the incorporation of structural information into GNN frameworks, which has been shown to support functional inference and offers a promising avenue for the fight against AMR by improving AMP prediction.^28,31–33^

A great example of the power of GNN-based architectures is the therapeutic peptide language model multi-scale graph neural network (TP-LMMSG), which propagates information across amino acids by modeling peptides as graphs, thereby accounting for non-Euclidean structural relationships (Table 1).^34^ By combining seven datasets and multiple feature representations, TP-LMMSG integrates language model embeddings, physicochemical descriptors, and structural encodings into a unified graph representation, achieving superior accuracy not only for AMPs but also for antiviral and anticancer peptides, while improving preprocessing and storage efficiency.^34^ Similar graph-based strategies have been adopted by sAMPpred-GAT,^33^ which applies graph attention networks (GAT) to capture residue-level interactions and contextual dependencies relevant to antimicrobial activity, leading to improved predictive performance compared to conventional sequence-based models (Table 1), and by PGAT-ABPp,^32^ which combines protein language model (PLM) embeddings with GAT to jointly model global sequence information and local structural features (Table 1). Collectively, these models demonstrate that explicitly encoding peptides as graphs enables a more effective representation of structural complexity and residue interactions, which are not easily captured by sequence-only approaches, thereby enhancing accuracy and robustness in AMP prediction.^32,33^

Nevertheless, graph-based architectures require careful preprocessing pipelines to construct and normalize graphs, which complicates reproducibility across laboratories. They are also computationally demanding and present interpretability challenges because of the high-dimensional latent representations they generate.^34^

The performance of AMP predictors is closely tied to the type of biological representation utilized. CNN-based models excel at detecting local motifs within embeddings, while RNN-based models effectively capture sequential dependencies across peptide chains, enabling the learning of long-range interactions that CNNs may miss.^21,27^ More recently, GNN-based methods have been introduced to encode molecular structures directly, offering representations that integrate both topological and spatial molecular information beyond linear sequence data.^21,34^ Comparative benchmarking further reveals that the choice of model architecture has significant effects on classification accuracy, precision, and false-positive rates.^20,35^ Each architectural approach brings unique advantages but also bears inherent limitations that must be evaluated when developing reliable pipelines for AMP discovery.^19,31^

Comparative studies have demonstrated that hybrid models combining CNN and RNN components can outperform individual architectures when trained on sufficiently large and appropriate datasets, indicating that their complementary strengths may help overcome some limitations inherent to CNN-only or RNN-only predictors.^19,27^ DL models offer advantages for peptide prediction; however, their effectiveness strongly depends on data representation and careful dataset curation. Meanwhile, traditional ML approaches with well-engineered features remain competitive under specific conditions.^19^ Broader reviews emphasize that although GDL and large language models represent promising avenues, challenges persist, including issues of reproducibility, interpretability, dataset biases, and the disparity between computational predictions and experimental validation.^21,24^

#### 2.1.2 Other Predictors

The application of AI models has extended *in silico* peptide evaluation beyond binary antimicrobial classification to include quantitative predictions of efficacy and safety, which are directly relevant to tackling AMR.^36–38^ MIC prediction constitutes a key advancement because it provides a direct measure of antimicrobial potency and enables more informed prioritization of peptide candidates prior to experimental validation.^36,39^ In this context, EIPpred addresses the prediction of inhibitory peptides against *Escherichia coli* (*E. coli*) by formulating the MIC estimation as a regression task based on peptide sequence features (Table 1). The study evaluates ML regressors and identifies random forest (RF) as the most effective model for associating peptide sequence features with lower MIC values in the curated dataset. By relying on conventional ML rather than complex architectures, EIPpred adopts a computationally efficient strategy suitable for MIC-oriented peptide screening. However, the dependence on organism-specific data and sequence-level representations limits the broader generalization of these models to other pathogens.^36^ To address data scarcity, BERT-AmPEP60 ^39^ employed a transfer learning framework by fine-tuning the pretrained PLM ProtBERT,^40^ originally trained on large-scale UniProt/UniRef databases, for AMP analysis (Table 1). The model was adapted to predict MIC values for *E. coli* and *Staphylococcus aureus* (*S. aureus*), representing Gram-negative and Gram-positive pathogens with high clinical relevance. Importantly, the ATT mechanisms embedded in the bidirectional encoder representations from transformers (BERT) architecture enable the identification of amino acid positions that strongly influence antimicrobial activity.^39^ This interpretability allows researchers to move beyond black-box predictions and directly associate sequence features with functional relevance. Such information is particularly valuable for guiding rational peptide optimization against drug-resistant pathogens. By supporting prioritization and informed redesign of AMP candidates, BERT-AmPEP60 contributes to more efficient strategies to combat AMR.^39^

Beyond the quantification of antimicrobial activity, safety-related properties represent a major bottleneck in AMP development and must be addressed early in the design pipeline.^37,38^ A predictor for hemolytic activity, which reflects the tendency of peptides to disrupt host red blood cell membranes, has been constructed using a hybrid Transformer-CNN architecture that predicts hemolysis directly from peptide sequences (Table 1).^37^ In this framework, convolutional layers extract local sequence information by learning patterns formed by neighboring amino acids, thereby capturing the short-range dependencies that contribute to hemolytic activity. These features describe how adjacent residues interact along the peptide chain. Complementarily, Transformer layers operate on the entire sequence through ATT mechanisms, enabling the representation of relationships between amino acids that are distant in sequence but jointly influence the hemolytic profile. The integration of local and global sequence representations supports discrimination between hemolytic and nonhemolytic peptides.^37^ Although effective for early safety screening, hemolysis alone does not fully describe peptide toxicity. To address this limitation, ToxGIN introduces a structure-aware toxicity prediction model based on graph isomorphism networks (GIN) that integrates peptide sequences with computationally predicted three-dimensional structures (Table 1).^38^ By representing peptides as graphs and explicitly modeling spatial residue–residue interactions, ToxGIN captures toxicity related features that are not accessible to sequence-only models. This strategy improves toxicity prediction at the cost of increased computational complexity and dependence on the accuracy of structure prediction methods.^38^

In recent years, AI approaches have also been successfully applied to predict AMR to conventional antibiotics. Due to the large volume of data generated by related genetic methods, ML and DL techniques have become invaluable in addressing this crisis.^4,41^ In a recent study, multiple ML models have been used to predict *E. coli* resistance to four antibiotics, including ciprofloxacin, cefotaxime, ceftazidime, and gentamicin, using whole-genome sequencing data and three encoding methods to computationally represent the genome.^42^ In that work, the authors demonstrated that all models could predict AMR with an area under the curve (AUC) of up to 0.90. Also, the authors showed that the models applied could identify potential single-nucleotide polymorphisms (SNPs) and corresponding genes associated with resistance.^42^

Additionally, researchers have developed a bioinformatics tool named variant mapping and prediction of antibiotic resistance (VAMPr)^43^ based on an extreme gradient boosting tree algorithm (Table 1). The VAMPr maps genomic data to predict antibiotic resistance based on genetic variants. VAMPr achieves high predictive accuracy, with values for the AUC and receiver operating characteristics curve (ROC) values up to 0.90, indicating excellent predictive capability for antibiotic resistance in various bacterial species, including *E. coli* and *S. aureus*, confirming known mechanisms and identifying potential new ones.^43^

ML and DL approaches have significantly advanced antibiotic resistance prediction studies, contributing to the development of new treatment alternatives and facilitating the identification of key genetic drivers of resistance.^44^ In the realm of AMPs, AI-based strategies have shown promise in predicting inhibitory activities, assessing binding affinities in protein–peptide interactions, and evaluating toxicity.^24^ However, to the best of our knowledge, the application of AI tools to study or predict resistance mechanisms specific to AMPs remains unexplored. This suggests that similar to their role in antibiotic research, AI approaches could provide valuable insights into AMP resistance mechanisms.^24^

### 2.2 Generative Models

While predictive models typically rely on supervised learning and are primarily designed for data classification, generative models are generally trained through unsupervised approaches and focus on reconstructing data from a low-dimensional space to generate novel and highly realistic artificial samples.^25,28,45^ The following sections examine the two most widely used generative models, variational autoencoders (VAEs) and generative adversarial networks (GANs), and provide a comparative discussion of their underlying principles and applications.

#### 2.2.1 Variational Autoencoder

VAEs are generative models that integrate autoencoders with elements of variational inference. They learn representations from unsupervised input data and generate new samples that resemble the training data. VAEs consist of key components, including an encoder network that maps inputs to a low-dimensional and probabilistic latent space based on a set of potential representations for each data point,^46^ a decoder network that reconstructs inputs from samples taken from this latent space, and a loss function aimed at minimizing the difference between the original and reconstructed inputs (Figure 2). The training process involves optimizing the parameters of both networks while constraining the latent space to approximate a standard Gaussian distribution, enhancing the model’s generalizability. Due to its ability to explore the entire dimension of latent space, VAE is posed to represent a wide range of features. Once trained, the model can manipulate data and produce diverse and realistic synthetic samples by rebuilding the latent space distribution through the decoder, making them particularly effective at capturing structured and interpretable representations. This property allows interpolation between data points and supports controlled sequence optimization and generation under stable training dynamics. By learning the underlying data distribution, these models can also sample novel peptide sequences, enabling the design of diverse AMP candidates while maintaining desirable functional properties.^24,46–49^

**Figure 2:**
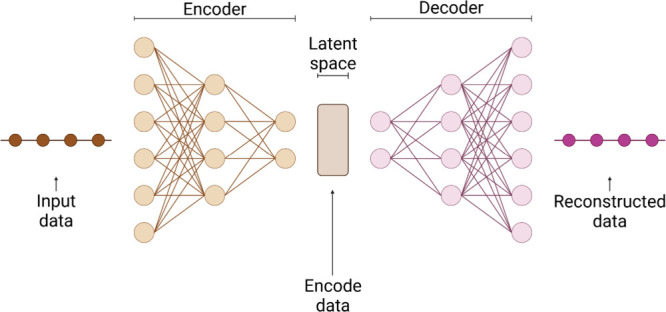
Structure of a VAE used for processing data related to AMPs. The model consists of an encoder, which compresses input data into a latent space, and a decoder, which reconstructs the original data from this latent representation. This figure was created with BioRender.com.

A prime example of the capabilities of VAE architecture is a study that trained the model on thousands of labeled and unlabeled peptide sequences to discover two novel peptides (YI12 and FK13) and evaluate their antimicrobial activity in just 48 days (Table 1).^50^ Both peptides proved effective against Gram-negative and Gram-positive bacteria, including multidrug-resistant (MDR) and polymyxin-resistant strains. In terms of therapeutic application, FK13 may exhibit greater biocompatibility than YI12. Moreover, both peptides displayed acceptable toxicity rates for therapeutic use and a low likelihood of resistance induction.^50^

VAE models have also been successfully applied to molecular optimization tasks. A notable example is a pioneering study that developed the query-based molecule optimization (QMO) framework, a versatile algorithm capable of optimizing molecules in discrete spaces, such as sequences or graphs (Table 1).^51^ Leveraging the encoder-decoder architecture of VAEs, this framework addresses the challenge of large molecular search spaces by encoding molecular sequences into low-dimensional representations. Its effectiveness was demonstrated by optimizing 150 known toxic AMPs from public datasets into 109 nontoxic variants.^51^ Using a different strategy, the multiobjective optimization by quantum annealing (MOQA) pipeline combines a binary VAE (bVAE), a variation of the traditional VAE that uses a binary vector to represent the latent space instead of a real-valued vector, and a D-Wave quantum annealer to design peptides based on multiple properties (Table 1).^52^ Trained on a dataset of over 25,000 AMP and non-AMP sequences, MOQA successfully generated 200,000 peptide sequences. Among these, four were selected for wet-lab validation, confirming the pipeline’s efficacy. Notably, peptides TA2-1, TA2-2, and TA2-3 exhibited high antimicrobial activity, with TA2-3 demonstrating the lowest MIC value among them.^52^

#### 2.2.2 Generative Adversarial Networks

Generative adversarial networks (GANs) have proven effective in designing peptides with tailored characteristics, such as antimicrobial, anticancer, and immunogenic properties.^18^ GAN consists of two main components: a generator and a discriminator. The generator is a neural network that takes random noise as input and produces data samples, learning to create increasingly realistic outputs that resemble the training data as the training progresses. Conversely, the discriminator is another neural network that evaluates input samples and predicts whether they are real or generated. For AMR, the GAN works by generating new peptide sequences, and a discriminator, which evaluates the authenticity of the generated sequences compared to real AMPs, similar to an AMP predictor (Figure 3).^24,28^

**Figure 3:**
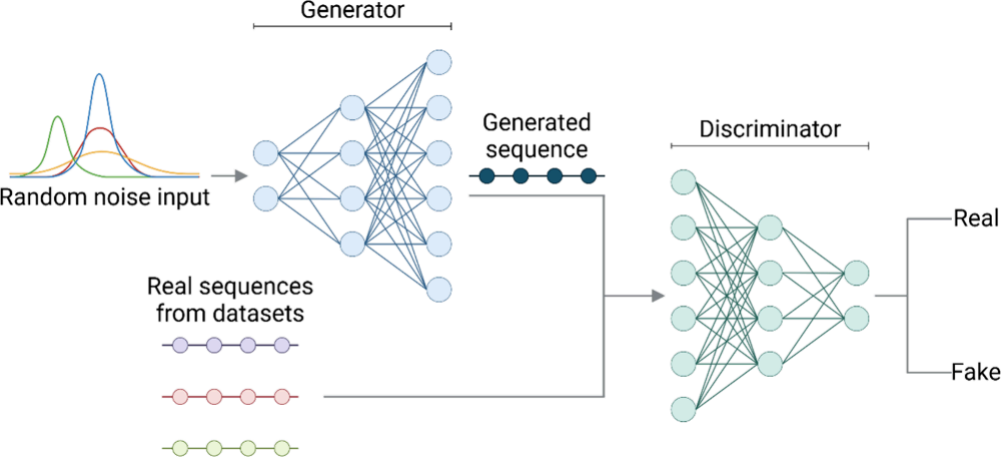
Structure of a GAN applied to the generation of AMPs. The GAN consists of two main components: a generator, which creates new peptide sequences, and a discriminator, which evaluates the authenticity of the generated sequences compared with real ones. This figure was created with BioRender.com.

The training process, known as adversarial training, involves both networks being trained simultaneously with the generator striving to create indistinguishable samples from real data, whereas the discriminator works to accurately classify real and fake samples. This iterative process continues until the generator produces outputs that the discriminator can no longer distinguish from real data. However, this process is highly susceptible to training instability and mode collapse, where the generator fails to produce diverse outputs and the adversarial training dynamics can lead to oscillations or failures.^25,46^ When training is successful, these competing dynamics converge toward a Nash equilibrium, where the generator’s distribution aligns with that of the training data, resulting in a discriminator that is maximally confused and unable to differentiate between real and fake samples.^25,28,53^

Multi-CGAN^54^ is a multilayer conditional GAN (CGAN) designed to generate peptides with desired properties by incorporating additional conditions into the generation process (Table 1). CGANs enable control over the generation process by adding specific conditions, ensuring that the output aligns with predefined criteria. In a multilayer CGAN, multiple conditions can be simultaneously applied, and only realistic sequences meeting all specified conditions are accepted by the discriminator. The Multi-CGAN model was trained to consider three distinct properties: (I) antimicrobial activity, (II) toxicity, and (III) secondary structure. When evaluated for its ability to generate realistic sequences, Multi-CGAN achieved the following accuracy rates: 90.9% when combining antimicrobial activity and toxicity, 88.8% for antimicrobial activity and secondary structure, 82.1% for toxicity and secondary structure, and 86% when all three layers were combined.^54^

VAEs and GANs present complementary advantages in the generation of synthetic biological data. Both architectures rely on a low-dimensional and probabilistic space to capture complex features of the input data and enable the creation of novel sequences, a property that underpins their capacity to expand the accessible design space for AMPs.^25,46^ Taken together, VAEs provide robustness and interpretability, whereas GANs deliver superior realism at the cost of training complexity. The integration of both approaches represents a promising avenue for accelerating the design and optimization of novel AMPs. GANs are also recognized for their ability to generate highly realistic synthetic data that closely mimic the training data distribution. This synthetic data can supplement limited AMP datasets and be combined with existing samples, making GANs valuable for data augmentation and for addressing the privacy concerns and scarcity of experimentally validated AMP data during ML training.^25,46^

## 3 Integration of AI with MultiOmics Data for AMP Mining and Discovery

Technological advances in data generation across various biological systems such as DNA sequencing, RNA expression, methylation patterns, epigenetic markers, proteomics, and metabolomics have propelled the field of translational bioinformatics over the past decade, driving a surge in data and the development of complementary analytical tools.^55^ In this context, the growth of “omics” fields and technologies has significantly enhanced drug screening by enabling tools that connect phenotypes to genotypes.^56^

Curated databases are essential for the effective analysis of nucleotide and protein sequencing data generated by these diverse platforms.^57^ As data acquisition grows, resulting in increasingly large and complex datasets, ML and AI have emerged as powerful alternatives to support effective analysis and data mining (Figure 4).^57^ AI algorithms are particularly valuable for integrating multiomics datasets, enabling the comparison and identification of patterns across vast quantities of biological data. This capability provides insights into complex cellular mechanisms, predicts clinical outcomes, and supports efficient drug design. Various methods for integrating multiomics data have been proposed, categorized as supervised, semisupervised, or unsupervised learning approaches.^58^

**Figure 4:**
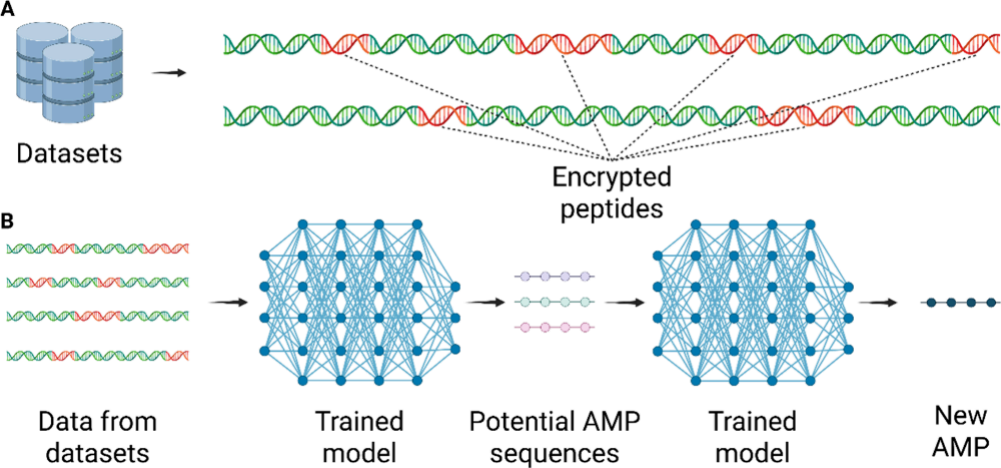
Identification of encrypted peptides and prediction of AMPs from genomic data. (A) Representation of a genomic database containing multiple genomes with encrypted peptides highlighted. (B) These genomic data are processed as input into an AI model, which identifies possible encrypted peptide regions and generates new peptide sequences with potential antimicrobial activity. This figure was created with BioRender.com.

An example of omics integration in drug design is a study where researchers explored and mined AMPs from the human gut microbiome.^8^ To address the challenge posed by microbiome diversity, the team developed advanced pipeline leveraging (NLP) and neural network models. Initially, they employed LSTM networks for AMP identification and incorporated an ATT model to analyze the relationships between amino acids in the peptides (Table 1). They also used the transformer-based model, BERT, to process the sequences as text, treating each amino acid as a word. The models were trained using a curated dataset of 10,322 experimentally validated AMPs and 3,029,894 non-AMP sequences, which were used to train and evaluate NLP-based classifiers for AMP identification.^8^ The trained models were then applied to small open reading frames (sORFs) predicted from 4409 high-quality human gut microbial genomes, generating 211,759,711 nonredundant sORF sequences. Screening of these sORFs with the trained models yielded 20,426,401 putative AMP sequences, which were subsequently filtered using large-scale human gut metaproteomic data to retain only peptides with evidence of *in vivo* expression, resulting in 2349 expressed candidate AMPs. These candidates were further prioritized using correlation network analysis across 11,011 metagenomic samples, yielding 241 high-confidence AMPs, of which 216 were synthesized and 181 demonstrated antimicrobial activity,^8^ yielding a success rate of over 83%. The final model, combining LSTM, ATT, and BERT, achieved an impressive precision of 91.31%, highlighting 11 AMPs with exceptional efficacy against multidrug-resistant Gram-negative pathogens. These AMPs exhibited remarkable potency, with some showing MICs below 25 μM, making them strong candidates for combating resistant bacterial infections.^8^

In another study, an RF-based model algorithm was used to mine AMP sequences from 63,410 metagenomes and 87,920 high-quality microbial genomes (Table 1).^59^ This resulted in over 5 million genes and a collection of 863,498 nonredundant candidate AMP sequences, which were deposited in AMPSphere. All mined sequences underwent *in silico* quality validation. When tested with other AMP prediction systems, such as AI4AMP^60^ and AMPLify,^30^ 98.4% (849,703 peptides) of the AMPSphere sequences were predicted as AMPs, with 15% (132,440 peptides) being copredicted by all methods used.^59^ The authors selected 100 sequences for synthesis and tested them against 11 clinically relevant pathogens, where 63 AMPs completely eradicated the growth of at least one pathogen. *Acinetobacter baumannii* (*A. baumannii*), *E. coli*, and vancomycin-resistant *Enterococcus* strains exhibited higher susceptibility, whereas methicillin-resistant *S. aureus* was unaffected by the peptides. Notably, four peptides (I) cagicin-1, (II) cagicin-4, and (III) enterococcin-1 against *A. baumannii* and (I) cagicin-1 and (IV) lachnospirin-1 against vancomycin-resistant *Enterococcus faecium* showed MIC values as low as 1 μmol L^–1^, similar to the peptide antibiotic polymyxin B.^59^

The vast amount of data and the ability to uncover encrypted peptides (EPs) have positioned the field of proteomics as a promising alternative for discovering potential therapeutic agents. Proteolytic action on precursor proteins can release bioactive fragments, offering valuable inspiration for the development of novel therapeutic molecules targeting microorganisms such as MDR pathogens.^61,62^ Recently, the ML algorithm panCleave was employed to predict cleavage sites across the entire proteome (Table 1).^63^ This tool identified encoded AMPs within modern human secreted proteins as well as in the archaic proteomes of our closest extinct relatives, Neanderthals and Denisovans. The encrypted peptides showed anti-infective efficacy against *A. baumannii* in both skin and thigh infections, introducing molecular de-extinction as an innovative approach to revolutionizing antibiotic discovery.^63^

In another innovative approach, APEX was developed to explore and extract encrypted peptides from extinctome datasets (proteomes of extinct organisms).^64^ The APEX architecture consists of multiple neural network layers (Table 1). Using a hybrid strategy, RNN and ATT models were integrated to form an encoder neural network that extracted features from peptide sequences. These extracted features were then processed by two distinct fully connected neural networks (FCNN), the first predicted species-specific antimicrobial activities, whereas the second performed a binary classification to determine whether the peptides exhibited antimicrobial activity.^64^ Due to this complex neural network architecture, APEX successfully mined over 10 million encrypted peptides and predicted their antimicrobial potential, leading to the identification of 37,176 AMP candidates. Among these, 69 peptides were synthesized and tested, with 41 (59%) demonstrating notable antimicrobial activity.^64^ APEX was employed to predict encrypted AMPs not only from extinctome datasets but also from venomics datasets. More recently, venom protein sequences were used as input data, resulting in over 40 million encrypted peptide sequences (Table 1). After the prediction step, this number was reduced to 7379 candidates. Among them, 58 were synthesized and tested, with 53 (91%) exhibiting antimicrobial activity.^65^

Lastly, to overcome the limitations of the APEX model due to its inherently discriminative nature, the APEX generative optimization (APEX_GO_) framework was developed (Table 1). This enhancement allows the model to refine peptide sequences through an iterative generative process, integrating VAE and Bayesian optimization (BO).^66^ The optimization begins with the VAE decoder, which transforms latent space points into peptide sequences. A surrogate model then maps these latent space points to the MIC values predicted by APEX. Leveraging this surrogate model, the BO algorithm selects new latent points that are more likely to produce peptides with enhanced antimicrobial activity. This iterative refinement continues until peptides with optimized properties are identified.^66^ With this improvement, APEX_GO_ successfully utilized 10 peptides derived from the proteomes of extinct organisms to generate sequences with progressively lower predicted MICs as the optimization advanced. When compared to the MIC distributions of both the APEX training peptides and the template peptides, the optimized peptides exhibited a marked shift toward lower experimental MIC values, signifying enhanced predicted antimicrobial activity.^66^ A total of 100 optimized peptides were synthesized and evaluated for their antimicrobial properties. Among them, 86 demonstrated notable antimicrobial activity, leading to a success rate of 86%, which significantly exceeded the 59% hit rate obtained using APEX alone.^64,66^

To facilitate comparison across methodologies, Table 1 compiles the major AI-based models referenced in this perspective, categorizing them by application scope, predictive or generative performance, and architectural design.

## 4 Future Perspectives

As highlighted in this perspective, ML and DL methods have become increasingly popular tools in the field of AMR, offering new opportunities to address the urgent need for alternative treatments. Although AMR is a natural phenomenon driven by the biochemical and genetic characteristics of bacteria,^67^ it is amplified by multiple interconnected factors, including the widespread use of antibiotics, evolutionary pressures, and human behavior, making it a pressing global challenge.^41,68^ Furthermore, through horizontal gene transfer mechanisms like conjugation, transformation, and transduction, they can acquire and disseminate antimicrobial resistance genes (ARGs), enhancing their adaptability and accelerating the evolution and spread of AMR.^68,69^

Given this scenario, the quick and accurate AMR mechanism identification methods will enable the discovery of new ways to overcome this situation.^22^ Phenotypic characterization remains the gold standard for traditional antimicrobial susceptibility testing (AST).^42,70^ However, molecular methods for determining the AMR have garnered significant attention. These methods deliver faster results and enable the detection of specific ARGs and mutations, offering valuable insights into the dissemination and evolution of AMR.^41,70^ As more data are generated across multiple data types and multiple tissues, novel explorations will assist our understanding of important biological processes and enable more comprehensive systems and genomic strategies,^55^ which could give us enough data to train AI models to predict AMR to AMPs and use these prediction data to reduce the likelihood of resistance to new antibiotics. Molecular generation methods have been improved in the past decade, and this trend is likely to continue for the next years. Computer technology and omics methodologies will advance even more with the assistance of AI in processing and storing data. Bearing this in mind, the data integration algorithms should take advantage of the big data era and the advent of ML and AI algorithms.^57,71^ Such advancements would not only improve our understanding and management of AMR related to AMPs but also play a pivotal role in designing peptides with optimized properties to counteract AMR.^24^

Building upon this foundation, integrating active learning frameworks with wet-lab feedback represents a transformative next step toward improving ML models in biological research. By iteratively selecting the most informative samples for experimental validation, these frameworks refine model accuracy and efficiency, facilitating more effective navigation of complex biological spaces while concurrently reducing experimental burden.^20,21^ By combining MIC with cytotoxicity and hemolysis assays, researchers can assess antimicrobial efficacy alongside safety-related properties, providing a more complete biological characterization that supports downstream translational development.^49^ Their clinical translation is also limited by instability and poor pharmacological behavior, predicting these properties and integrating them into AMP design pipelines provides valuable guidance for filtering candidates and directing rational amino acid substitutions.^39,49^

Drug delivery systems (DDS) are another strategy to support the clinical translation of AMPs by improving stability and reducing cytotoxicity.^72^ Computational approaches support the rational design of DDS by linking the physicochemical and structural characteristics of AMPs, such as length, hydrophobicity, topology, and energetic interactions, to their functional performance. Molecular dynamics simulations play a central role by enabling detailed analysis of AMP behavior in lipid bilayers and delivery systems, clarifying how peptide interactions affect stability, permeability, and release.^72^ These methods allow for rapid and cost-effective screening of peptide formulations, reducing the need for extensive experimental testing. However, these computational approaches must be integrated with *in vitro* and *in vivo* data to account for biological complexity and ensure translational relevance.^72^ While ML is widely used for AMP design, its application to DDS modeling remains limited, indicating the need for computational frameworks tailored to peptide delivery.^72^

Grounding computational predictions in empirical data improves both model reliability and interpretability, helping to mitigate the persistent black-box behavior of many deep learning approaches used in AI-driven AMP design.^49,73^ Model interpretability is critical for understanding the rationale behind predictions, such as identifying specific amino acid residues or motifs associated with activity or adverse effects, which is essential for guiding rational optimization. The lack of transparent reasoning in many deep learning architectures hinders the extraction of general design principles and limits the ability of researchers to learn from model outputs.^49,73^ Moreover, insufficient mechanistic insight complicates regulatory evaluation and slows clinical translation, where an explainability and safety understanding are required. Future advances will depend on the availability of larger, high-quality datasets and the deeper integration of explainable AI approaches into predictive pipelines, enabling actionable biological insight and increased trust in AI-driven AMP discovery.^49,73^ This methodology inherently promotes interdisciplinary collaboration between computational scientists and experimental biologists, fostering a shared understanding crucial for meaningful biological insights.^20,21^

Although challenges remain, such as optimizing sample selection strategies and managing associated experimental costs, the synergy of active learning and wet-lab validation promises to accelerate the discovery of novel peptides, drugs, and biomolecules. Such integration holds great potential to advance bioinformatics and therapeutic design by enabling more targeted and validated predictions, ultimately expediting translational research and clinical applications.^24,50^

With high-quality data generation expected to become increasingly prevalent with the advancement of AI, developing effective strategies to train models on such data will pose a significant challenge. As previously demonstrated, selecting the most appropriate feature representation directly impacts the model’s final performance.^19^ Most feature representations have been designed for peptides composed of the 20 natural amino acids, often disregarding noncanonical or chemically modified residues during training. As a result, these models may struggle to generalize to nonstandard peptides.^20^ In this context, studies that simultaneously integrated multiple feature representations with various architectures achieved higher efficacy in their tasks.^34,64,66^ Despite their increased complexity due to multiple-layer processing, these models can simultaneously capture diverse peptide properties such as primary sequence, physicochemical characteristics, and three-dimensional structural information within a high-dimensional space. This capability addresses one of the key challenges in accurately representing such features in a trained model.^34,64,66^ This strategy could also serve as a potential solution to overcome the limitations posed by noncanonical and chemically modified amino acids.

However, as models and training strategies are expected to grow in complexity by accounting for an increasing number of parameters, the demand for higher computational power becomes a limiting factor, highlighting the need for improved computing systems capable of efficiently processing such data.^74^ In this context, quantum computing (QC), based on the principles of quantum mechanics, emerges as a groundbreaking approach.^75,76^ Unlike traditional computers that use bits to represent data in binary form (0 or 1), quantum computers use qubits, which can exist in a superposition of states, enabling them to represent both 0 and 1 simultaneously and to explore multiple possibilities in parallel, thereby offering unprecedented computational power.^75,76^ The processing capability of these chips could not only handle all amino acid variations with high accuracy but also account for their interactions with cellular metabolism.^76,77^

Nevertheless, QC still faces substantial hardware limitations, including qubit noise, limited coherence times, and high error rates, which compromise stable computations and hinder the transition to fault-tolerant operation. Although recent progress in error correction has been demonstrated, current qubit technologies remain affected by hardware noise, preventing the reliable operation required for large-scale fault-tolerant computing.^77–79^ In addition, scaling qubit numbers while preserving connectivity, fidelity, and efficient error correction remains a major challenge. Existing quantum processors lack sufficient qubits and architectural maturity to address large-scale problems, and leading platforms such as superconducting qubits, trapped ions, and topological qubits are still under active development.^77–79^ Achieving practical fault-tolerant quantum computing will require significant advances in qubit architectures, error correction strategies, and system-level engineering, suggesting that future applications will remain constrained. Consequently, realistic progress in QC is expected to depend on sustained interdisciplinary efforts spanning physics, engineering, AI, and biology.^77–79^ For a deeper understanding of AI in QC, we encourage reading the work from Alexeev et al., 2025.^79^

As quantum hardware, software, and algorithms advance, breakthroughs in predictive accuracy, structural optimization, and *de novo* peptide generation are becoming increasingly feasible.^74,78^ When integrated with AI and omics-based approaches, this technology will enable the *in silico* design of highly effective AMPs capable of evading bacterial resistance.^77,78^ QC is set to transform the design of AMPs by overcoming some of the most computationally intensive challenges in molecular biology.^80^ Unlike classical systems, which struggle to accurately capture the quantum nature of molecular interactions, quantum computers are inherently equipped to simulate quantum phenomena with high precision, which could offer a more accurate representation of chemical reactions and peptide-target binding.^76,80^ This capability is particularly crucial in AMP discovery, where understanding the precise folding and interaction of each amino acid determines biological efficacy.^78,81^ This convergence could mark the beginning of a new era in precision therapeutics, offering powerful tools to combat MDR pathogens.^78^
